# Developing mathematical model for diurnal dynamics of photosynthesis in *Saccharum officinarum* responsive to different irrigation and silicon application

**DOI:** 10.7717/peerj.10154

**Published:** 2020-10-27

**Authors:** Krishan K. Verma, Kai-Chao Wu, Chhedi Lal Verma, Dong-Mei Li, Mukesh Kumar Malviya, Rajesh Kumar Singh, Pratiksha Singh, Gan-Lin Chen, Xiu Peng Song, Yang Rui Li

**Affiliations:** 1 Key Laboratory of Sugarcane Biotechnology and Genetic Improvement (Guangxi), Ministry of Agriculture and Rural Affairs/Guangxi Key Laboratory of Sugarcane Genetic Improvement/Sugarcane Research Institute, Guangxi Academy of Agricultural Sciences, Nanning, Guangxi, China; 2 Central Soil Salinity Research Institute (RRS), Lucknow, Uttar Pradesh, India; 3 Institute of Biotechnology, Guangxi Academy of Agricultural Sciences, Nanning, Guangxi, China

**Keywords:** Biomass, Drought, Diurnal, Leaf gas exchange, *Saccharum officinarum* L., Silicon

## Abstract

In the dynamic era of climate change, agricultural farming systems are facing various unprecedented problems worldwide. Drought stress is one of the serious abiotic stresses that hinder the growth potential and crop productivity. Silicon (Si) can improve crop yield by enhancing the efficiency of inputs and reducing relevant losses. As a quasi-essential element and the 2nd most abundant element in the Earth’s crust, Si is utilized by plants and applied exogenously to combat drought stress and improve plant performance by increasing physiological, cellular and molecular responses. However, the physiological mechanisms that respond to water stress are still not well defined in *Saccharum officinarum* plants. To the best of our knowledge, the dynamics of photosynthesis responsive to different exogenous Si levels in *Saccharum officinarum* has not been reported to date. The current experiment was carried out to assess the protective role of Si in plant growth and photosynthetic responses in *Saccharum officinarum* under water stress conditions. *Saccharum officinarum* cv. ‘GT 42’ plants were subjected to drought stress conditions (80–75%, 55–50% and 35–30% of soil moisture) after ten weeks of normal growth, followed by the soil irrigation of Si (0, 100, 300 and 500 mg L^−1^) for 8 weeks. The results indicated that Si addition mitigated the inhibition in *Saccharum officinarum* growth and photosynthesis, and improved biomass accumulation during water stress. The photosynthetic responses (photosynthesis, transpiration and stomatal conductance) were found down-regulated under water stress, and it was significantly enhanced by Si application. No phytotoxic effects were monitored even at excess (500 mg L^−1^). Soil irrigation of 300 mg L^−1^ of Si was more effective as 100 and 500 mg L^−1^ under water stress condition. It is concluded that the stress in *Saccharum officinarum* plants applied with Si was alleviated by improving plant fitness, photosynthetic capacity and biomass accumulation as compared with the control. Thus, this study offers new information towards the assessment of growth, biomass accumulation and physiological changes related to water stress with Si application in plants.

## Introduction

Water scarcity is one of the most crucial abiotic stresses for plants in the dynamic era of climate change. A number of studies have mentioned that water deficit is more harmful than other abiotic stresses (Chen et al., 2017; Verma et al., 2019a; Li et al., 2019; Raza et al., 2019; Verma et al., 2020). Under limited or highly variable water, plants have developed various mechanisms of resistance to water deficit by reduction of the plant life cycle (Mitra, 2001; Cia et al., 2012). Therefore, plants have evolved in various cellular and molecular strategies to cope with water deficit (Ijaz et al., 2017; Ali et al., 2018). Abiotic stresses are estimated to reduce about 51–82% agricultural crop production. The metabolic changes to water deficit in varieties with various responses to water stress have been well documented in different agricultural crops (Kang et al., 2011; Witt et al., 2012; Silvente, Sobolev & Lara, 2012; Zhao et al., 2014; Verma et al., 2019a, 2020).

Low soil water capacity in the dry season is one of the most important limitations to photosynthesis and consequently to *Saccharum officinarum* production (Chen et al., 2011; Verma et al., 2019a). Under limited water conditions, disturbances in photosynthetic apparatus at the molecular and cellular levels are associated with low electron transport through photosystem II (PS II) and/or with structural damages of PS II and the light harvesting complexes (Hura et al., 2007; Wu et al., 2008). Photosynthetic and growth responses are dependent on environmental variables and developmental phages. It is expected that changes in temperature, light intensity and available water content, and changes across the growth phases will influence the growth and physiological dynamics during diurnal cycle (De Souza et al., 2018; Verma et al., 2019a). Improving photosynthetic capacity is linked for the enhancement of biomass and crop productivity. Soil water content and nutrients can also play significant roles in sustaining the photosynthetic responses in agricultural crops (Shangguan, Shao & Dyckmans, 2000; Li et al., 2016, 2019; Khan et al., 2017; Verma et al., 2020). Under water deficiency, Si application can improve and/or enhance photosynthetic capacity, root growth-development, nutrient uptake and consequently increase crop productivity (Verma et al., 2019a, 2019b, 2019c; Li et al., 2019). Various agronomic strategies have been adopted for this purpose. One of the advanced strategies is the use of plant bio-stimulator to enhance the adaptability and protection of crop plants subjected to environmental stresses.

Silicon (Si) is the second most important element in the earth’s crust, which can improve crop resistance to reduce the negative impacts of biotic and abiotic stresses such as insufficient water, extreme air temperature, UV, cold, alkalinity, nutritional imbalance, heavy metal toxicity, plant pathogens and insect pests in various crop plants (Liang et al., 2007; Guo et al., 2016; Reynolds et al., 2016; Ju et al., 2017; Chen et al., 2018; Verma et al., 2019a). Plants generally take up Si in the form of silicic acid from soil and nutrient solutions and Si is the only nutrient element that is not detrimental when absorbing excess in the plant’s organ (Ma, Miyake & Takahashi, 2001; Ma & Yamaji, 2006; Chen et al., 2018). The maximum solubility of Si(OH)_4_ in solution is nearly 2 mM, and its concentration in soil solutions usually differ between 0.1 and 0.6 mM (Raven, 1983; Epstein, 1994). Moreover, orthosilicic acid (p*K*a_1_ = 9.84, p*K*a_2_ = 13.2, at 25 °C), the form of Si accessible to plants (Casey et al., 2004), is soluble in water only up to about 2 mM at 25 °C, above which polymerization into silica (SiO_2_) gels begins to occur (Ma, Miyake & Takahashi, 2001). Under similar situations, plant varieties have different abilities to accumulate Si, a reality that has been known, if poorly understood, for a long time.

The protective role of Si in metabolic, physiological and/or anatomical activities in crop plants against environmental stresses have been widely documented (Van Bockhaven, De Vleesschauwer & Höfte, 2013; Zhu & Gong, 2014; Shi et al., 2016; Verma et al., 2019a). The beneficial effects of Si against limited water supply/water deficiency have been extensively assessed in many crop plants, like *Oryza sativa* (Ming et al., 2012), *Zea mays* (Kaya, Tuna & Higgs, 2006; Amin et al., 2014), *Triticum aestivum* (Pei et al., 2010; Gong & Chen, 2012), *Sorghum bicolor* (Liu et al., 2014), *Solanum lycopersicum* (Shi et al., 2016), *Saccharum* spp. (Verma et al., 2019a, 2019b, 2019c, 2020), cucumber (Ma et al., 2004), Kentucky bluegrass (Saud et al., 2014), canola (Habibi, 2014) and alfalfa (Liu & Guo, 2013).

Sugarcane (*Saccharum officinarum* L.) is one of the most important cash crop in the globe due to its great demand for sugar and renewable energy sources to replace fossil fuels. Unlikely, in various regions, especially in the tropical and sub-tropical areas, the production of *Saccharum officinarum* is markedly decreased up to 60% due to availability of insufficient water for irrigation (Robertson et al., 1999; Verma et al., 2019a).

However, knowledge about how Si modulates the morphological, physiological and biomass accumulation in *Saccharum officinarum* “GT 42” during water stress remains elusive. Although the essentiality of this element to plants is still debated, there have been significant impacts in our understanding of the uptake of Si in plants. In addition, the present database regarding the precise amount of Si for its application method in *Saccharum officinarum* plants is limited. Therefore, the present study was conducted to investigate the possible impacts of exogenous application of Si on growth, biomass accumulation and photosynthetic responses in *Saccharum officinarum* plants subjected to water stress. Our work in *Saccharum officinarum* may help to better understand the mechanisms and functions for Si-mediated water stress tolerance in plants.

## Materials and Methods

**Figure 1:**
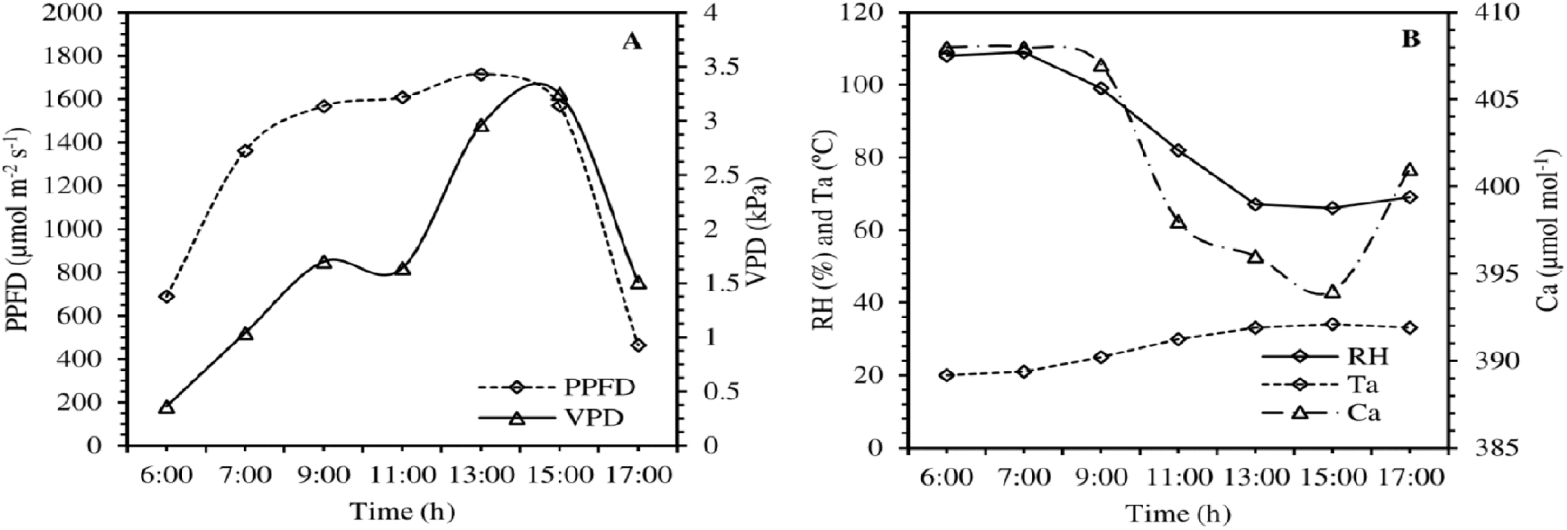
Diurnal changes of climatic variables such as photosynthetically photon flux density (PPFD), vapor pressure deficit (VPD), air relative humidity (RH), air temperature (Ta) and ambient CO_2_ concentration (Ca) from 06:00 to 17:00 (A and B) on the measuring day.

*Saccharum officinarum* “GT 42” single bud cane setts were planted in fertile farmland soil in greenhouse in March 2019 at Sugarcane Research Institute, Guangxi Academy of Agricultural Sciences, Nanning, Guangxi, China. After germination (8 weeks), the seedlings were shifted in pots (soil capacity 3.5 kg), and continued to receive full irrigation to keep moisture capacity at 100–95% for proper root development before treatment. For the stressed treatment, limited water was imposed by gradually withdrawing irrigation until 80–75%, 55–50% and 35–30% of soil moisture capacity, determined by Soil Moisture Meter (Top Instrument Co. Ltd., Zhejiang, China). At the end of July, silicon fertilizer was applied as 0, 100, 300 and 500 mg L^−1^, respectively, directly in the soil. Calcium metasilicate powder (Wollastonite, CaO.SiO_2_) was used as a source of Si. The irrigation water was applied to the plant roots to keep 100–95% (normal), 80–75% (mild), 55–50% (moderate) and 35–30% (severe) of soil moisture capacity, respectively. The total amount of Si solution applied to well water and stressed-plants was the same. The climatic variables (ambient air temperature (Ta), air relative humidity (RH), ambient CO_2_ concentration (Ca), photosynthetic photon flux density (PPFD) and vapor pressure deficit (VPD)) were recorded diurnally (Fig. 1). The experiment was designed as completely randomized with ten biological replicates.

### Diurnal gas exchange

Photosynthesis (*P*_*N*_), stomatal conductance (*gs*) and transpiration rate (*E*) were observed at 60 days after treatment with limited water and exogenous use of Si, using portable photosynthesis system (LI-COR 6800; Biosciences, Lincoln, NE, USA). Diurnally leaf gas exchange was measured every 2 h from 07:00 to 17:00 in cloudless day on stressed and non-stressed plants (five replicates). The physiologically active leaves (middle part of leaf +1) were selected to place the leaf chamber for measuring all photosynthetic parameters at open environmental conditions without changing leaf angle/position.

### Determination of morphological and biomass traits

The plant height and leaf area-expansion were observed by a measuring meter and Leaf Area Meter (CI-203 Area Meter; CID, Inc., Camas, WA, USA). At the end of experiment (60 days), the *Saccharum officinarum* plants were harvested, washed with running water and weighted. The plant organs were kept in paper bags, oven-dried at 65 ± 2 °C and dry mass was weighted until the weight was constant.

### Model hypothesis

Physiological responses of plants are a result of complex chain reactions. Solar radiation is an essential input to start *P*_*N*_ and other associated bio-enzymatic chemical reactions. E and *gs* are interrelated with *P*_*N*_ process. Initially with increase in solar radiation *P*_*N*_ increases and acquires the highest optimum *P*_*N*_. Soon after initiations of *P*_*N*_ process inhibitive internal chain reactions also get started resulting in decline of *P*_*N*_ and interrelated bio-enzymatic chemical chain reactions within the leaves even after steady increase in solar radiation. Higher temperature is an indicator of high kinetic energy which speeds up chemical reactions in addition to initiate the process. After an optimum temperature level the enzymes start becoming denatured retarding the *P*_*N*_ process. For different plant species optimal temperature requirements differ (Herrmann, Haeder & Ghetti, 1997). Increase in solar radiation causes increase in temperature of the surrounding affecting the *P*_*N*_. Thus *P*_*N*_ and related physiological responses are function of climatic parameters such as PPFD, RH, Ca and Ta.

Figures 1A and 1B shows the diurnal variations of PPFD, Ta, Ca, RH and VPD. The variation of PPFD is skewed. From an initial zero value (5:00) it reaches the peak between 9:00 and 13:00 and thereafter it starts declining continuously to a zero value (19:00) at sun set. It can be further seen from Fig. 1B that the temperature starts rising immediately after sunrise and reaches a peak value between 13:00 and 15:00 and starts declining thereafter. The sun set temperature is much higher than the sun rise temperature during the day of observation. The continuous reduction of relative air humidity with sun shine hour (Fig. 1B). Air humidity was observed to be 88% at 6:00 in the morning and declines to the tune of 36% at 17:00 in the evening. RH is minimum at 15:00 and starts increasing thereafter. The leaf temperature changes with time which follows the suit of Ta. Initially it remains higher but immediately after acquiring the peak it becomes lower than the ambient. The overall physiological response variation is determined by the pattern of solar radiation. It directly affects the rate and pattern of physiological responses. Before acquiring the photosynthesis peak rate, retarding and inhibitive processes also starts, which slow down the *P*_*N*_ and other physiological responses. Biochemical changes taking place within the cell during the *P*_*N*_ affect the physiological responses. Overall physiological responses are dependent on direct responsive factors such as solar radiation and inhibitive and retarding factors inside the plant cell and climatic conditions of the plant leaves such as decreasing solar radiation, increasing air and leaf temperature and depleting RH and their effect on physiological responses of plant cell. Change of physiological responses with respect to time can be hypothesized as below.

Changes of physiological parameters are directly proportional to summation of *n*^th^ order responsive physiological response rate (*p*/*t^n^*). Mathematically it can be written as:

(1) $$\displaystyle{{dp} \over {dt}}\,\, \propto \sum\limits_{n = 1}^n {\displaystyle{{{\rm{\kappa}} \,p} \over {{t^n}}}}$$

where,

*p* = physiological parameters

*t* = time

κ = order of the physiological parameter’s constant

*n* = order of the rate of physiological parameters

Eq. (1) can be expanded as below.

(2) $$\displaystyle{{dp} \over {dt}}\,\, \propto \,\left( {\displaystyle{{{\rm{\alpha}} .p} \over t}\, + \,\displaystyle{{{\rm{\beta}} .p} \over {{t^2}}} + \displaystyle{{{\rm \gamma} .p} \over {{t^3}}} + \cdots + \displaystyle{{{\rm{\mu}} .p} \over {{t^{n - 1}}}} + \displaystyle{{\rm \xi .p} \over {{t^n}}}} \right)$$

where,

α, β, γ … μ and ξ = individual order constants

Considering *n* = 2 and ignoring the higher terms on Eq. (1) reduces to the following form.

(3) $$\displaystyle{{dp} \over {dt}}\,\, \propto \,\left( {\displaystyle{{{\rm{\alpha}} \,p} \over t}\, + \,\displaystyle{{{\rm{\beta}} p} \over {{t^2}}}} \right)$$

Equation (3) can be now rewritten as below:

(4) $$\displaystyle{{dp} \over {dt}}\,\, = \, {\rm{\lambda}} \,\,\left( {\displaystyle{{{\rm{\alpha}} \,p} \over t}\, + \,\displaystyle{{{\rm{\beta}} p} \over {{t^2}}}} \right)$$

where λ is proportionality constant.

Separating variables and integrating above equation one will obtain.

(5) $$\int {\displaystyle{{dp} \over p}} \,\, = \,\lambda \,\int {\,\left( {\displaystyle{{\rm \alpha \,} \over t}\, + \,\displaystyle{\rm \beta \over {{t^2}}}} \right)dt}$$

(6) $$\ln p = \,\lambda \left[ {\rm \alpha \,\ln \,t - \,\displaystyle{\rm \beta \over t}} \right] + C$$

(7) $$\ln {p_o} = \, {\rm{\lambda}} \left[ {{\rm{\alpha}} \,\,\ln \,{t_o} - \,\displaystyle{{\rm{\beta}} \over {t_o^{}}}} \right] + C$$

(8) $$C\, = \,\ln \,{p_o}\, - \left[ {{\rm{\alpha}} \,\ln \,{t_o} - \displaystyle{{\rm{\beta}} \over {{t_o}}}} \right]\, = \, {\rm{\psi}}$$

In Eq. (8) all the terms are constants which were replaced by another constant “ψ” and substituting the value of *C* = ψ into Eq. (6) one will get general solution of Eq. (4).

(9) $$\ln p = \,{\rm{\lambda}} \left[ {{\rm{\alpha}} \,\ln \,t - \,\displaystyle{{\rm{\beta}} \over t}} \right] + {\rm{\psi}}$$

Taking antilog on the both side the solution can be rewritten as under:

(10) $$p = \,{e^{\big[ {{\rm{\alpha}} \,\ln \,t\, - \,{{\rm{\beta}} \over t}} \big]{\rm{\lambda}} \, + {\rm{\psi}} }}$$

Combining all the constant terms together the above equation will take the following forms:

(11) $$p = \,\,{e^{\big[ {{\rm{\omega}} \,\ln \,t\, - \,{{\rm{\eta}} \over t}} \big]\, + {\rm{\psi}} }}$$

where, ω = αλ and η = βλ.

The model cannot be defined at time *t* = 0, hence should be started with some opening value of physiological response against a given time.

### Verification of the model

Physiological parameters such as *P*_*N*_, *E* and *gs* under drought stressed and normal conditions with Si application were fitted in the derived models (Eq. 10) and their parameters were worked out (Fig. S1; Table S1).

### Cumulative photosynthetic responses

Cumulative responses are essential for assessing the performance of plants under limited water irrigation. Integration of Eq. (10) is difficult hence its numerical integration was obtained. Graphical integration is easier and can be used for field application. Cumulative photosynthetic responses were calculated numerically by integrating proposed model and presented in Tables S2–S4 and variations are shown in Figs. S2–S4.

(12) $${P_t} = \,\int\limits_o^t {{e^{\big[ {{\rm{\mu} \,\ln \,t\, - \,{{\rm{\eta}} \over t}} \big]\, + {\rm{\xi}} }}dt }}$$

where,

*P_t_* = cumulative photosynthetic response from time *t* = 0 to time *t* = *t*.

Daily total photosynthetic CO_2_ assimilation (178.74–200.65 (control), 143.32–159.34 (mild), 111.25–120.21 (moderate) and 86.04–91.74 µmol CO_2_ m^−1^s^−1^ (severe stress)), transpiration rate (24.99–28.47 (control), 22.56–24.50 (mild) 19.41–20.89 (moderate) and 15.78–17.14 mmol CO_2_ m^−1^s^−1^ (severe stress)) and stomatal conductance (1,353.90–1,530.89 (control), 1,070.48–1,270.27 (mild), 746.78–867.65 (moderate) and 537.60–660.69 mmol H_2_O m^−2^ s^−1^ (severe stress)) were observed under limited water irrigation and Si application. There was almost 50% gain in photosynthetic parameters due to limited water and Si. It can be further seen from Tables S2 to S4 and Figs. S2 to S4 that by 12:00 at noon almost 60% photosynthetic responses are achieved and in the afternoon nearly 40% responses are achieved.

The experimental data were organized and processed between the limited water and Si application (± SD, *n* = 5). Data were analyzed by using GraphPad Prism 5.00 statistical software for windows (GraphPad Software, San Diego, CA, USA). One-way analysis of variance (ANOVA) was carried out to find out the significant differences among the treatment means at *P* < 0.05.

## Results

### Diurnal variation of environmental variables

On the measurement day, the PPFD enhanced steeply from 06:00 to 11:00, remained at highest levels up to 15:00, and then decline sharply. Under the impacts of PPFD diurnal changes, Ca and RH (Fig. 1) were at maximum in the early morning, followed by a sharp decline, remaining at relatively low levels during the midday period, and then began to enhance from 15:00. In contrast, Ta exhibited a diurnal trend similar to PPFD (Fig. 1).

### Effect of silicon on growth and biomass traits

**Figure 2:**
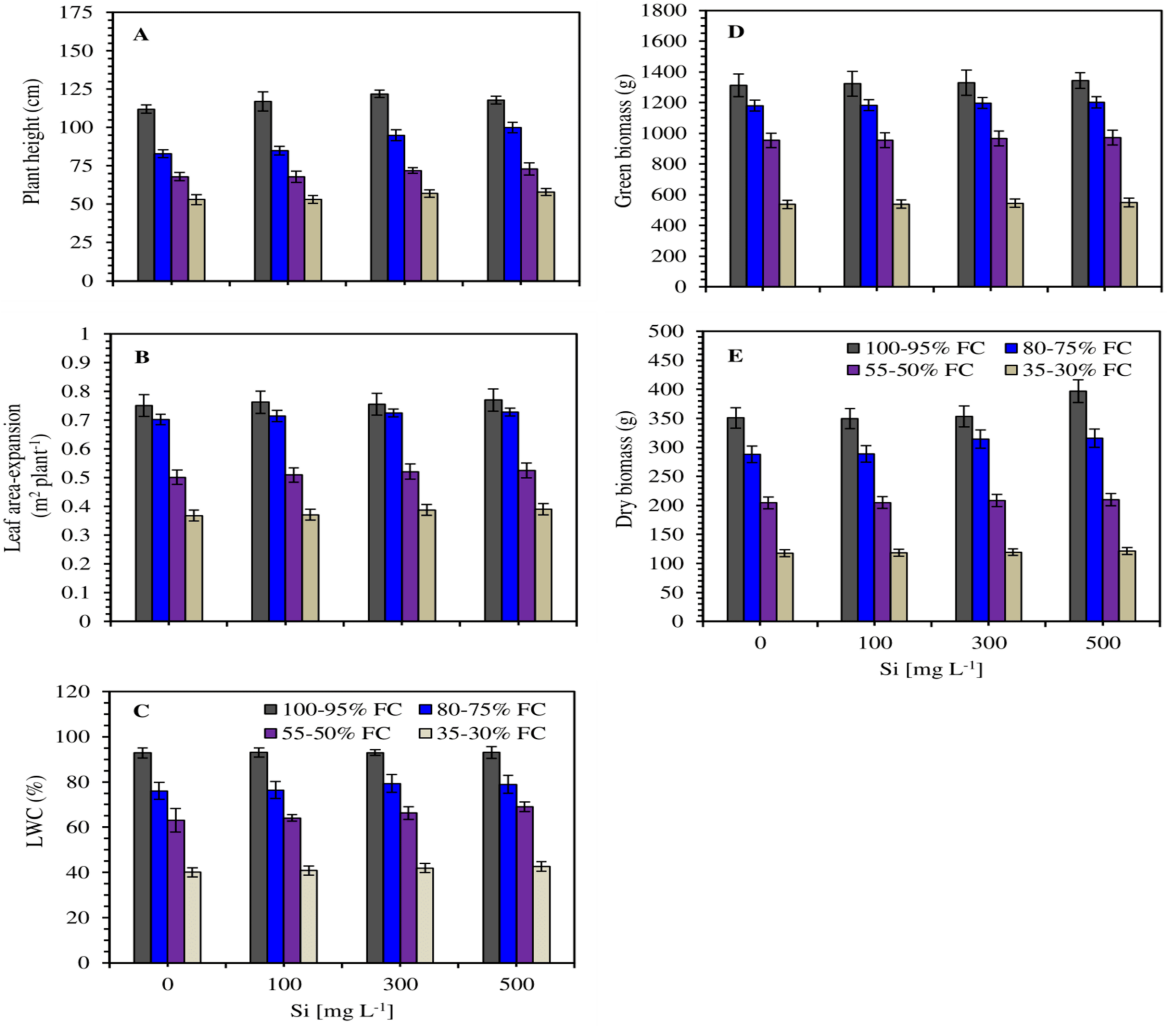
Effect of silicon (0, 100, 300 and 500 mg L^−1^) on plant height (A), leaf area-expansion (B), leaf water content (LWC) (C), fresh/green (D) and dry mass (E) in *Saccharum officinarm* plants against limited soil moisture capacity (100–95%, 80–75%, 55–50% and 35–30% of FC). Data are means ± SD (*n* = 5). FC = field capacity.

The *Saccharum officinarum* plants showed a drastic decline in plant height (PH), leaf area-expansion (LAE), leaf relative water content (LWC) and plant biomass (fresh and dry) under water stress, compared to well irrigation control (Figs. 2A–2E). The negative effects of low soil moisture capacity on growth and biomass were significantly (*P* < 0.05) mitigated and gradually enhanced with increasing Si levels (100–500 mg L^−1^). PH, LAE, RWC, fresh and dry mass of the plants in 100–95%, 80–75%, 55–50% and 35–30% of soil moisture capacity were found higher than the control without Si, with 2.41–20.48%, 0.65–6.12%, 0.17–9.54%, 0.14–2.42% and 0.30–13.12% increase, respectively (Figs. 2A–2E).

Growth and biomass traits exhibited an initial increase with the application of Si (100–300 mg L^−1^) and then declined considerably at excess (500 mg L^−1^). Shoot fresh and dry biomass accumulation in Si-treated plants showed significant (*P* < 0.05) improvement compared to normal plants under stress condition. The 300 mg L^−1^ Si concentration exhibited the most significant effects on growth and biomass of *Saccharum officinarum* plants, followed by 500 mg L^−1^ Si (Fig. 2).

### Water limitation and silicon effects on the diurnal changes of leaf gas exchange

**Figure 3:**
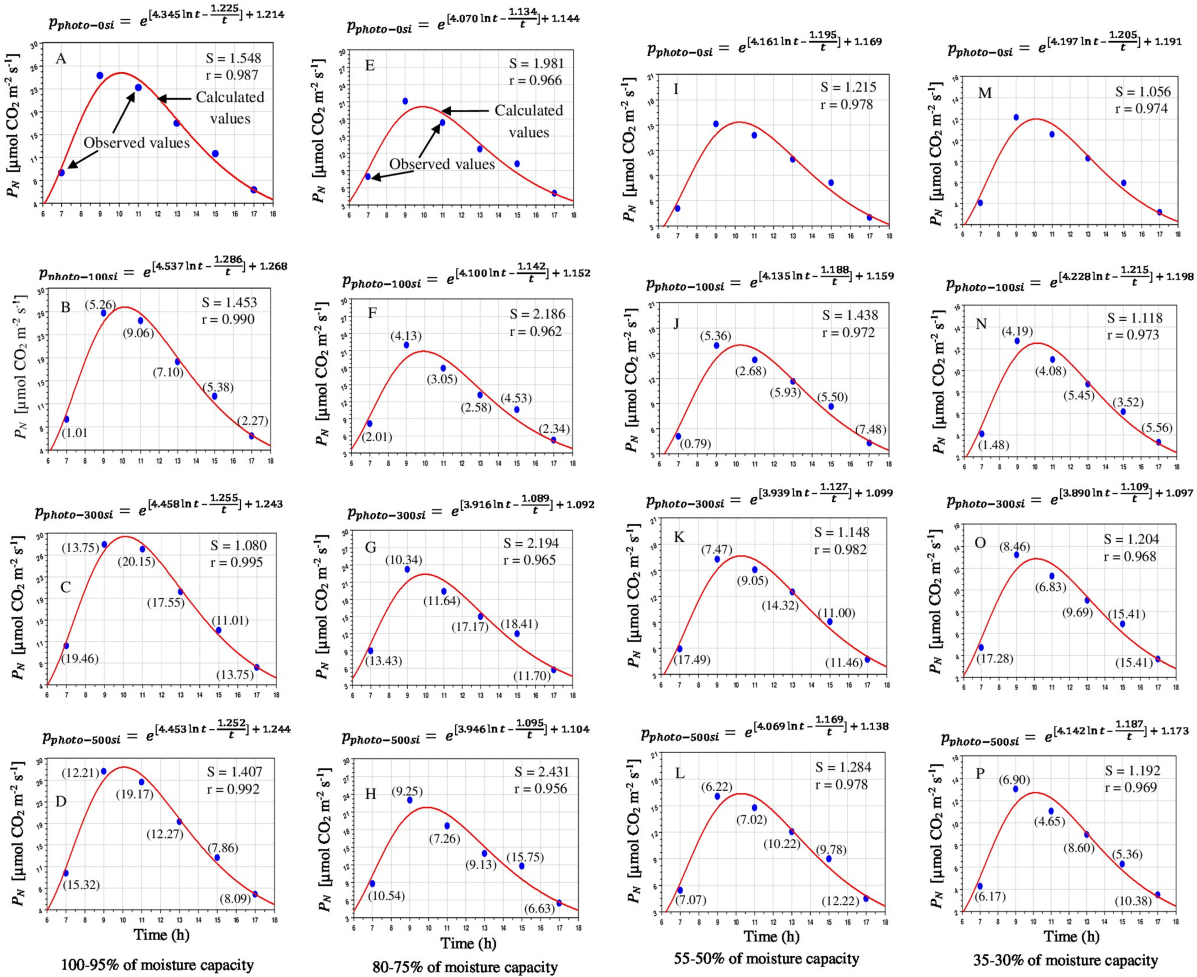
Diurnal effects of silicon on photosynthetic CO_2_ assimilation rate in *Saccharum officinarm* “GT 42” plants under limited water supply such as 100–95% (A–D), 80–75% (E–H), 55–50% (I–L) and 35–30% of soil moisture capacity (M–P) with four levels of Si concentrations, for example, 0, 100, 300 and 500 mg L^−1^. Parenthesis values indicate percentage gain against control condition.

One of the prime impacts of water stress is on the physiological process of photosynthetic responses. Water stress caused a severe loss in photosynthesis. Diurnal changes of photosynthesis are shown in Fig. 3. Overall the pattern of *P*_*N*_ mirrored that of *gs*. *P*_*N*_ and *gs* maximum reached at 9:00 as PPFD and *gs* enhanced. Subsequently, *P*_*N*_ steadily declined in all stressed plants and Si supplemented until a minimum value was reached in the early evening (17:00), similar to *gs* and *E*. Hence, *gs* was the main limiting factor for *P*_*N*_ of mesophyll cells at this time of the day. At 17:00, photosynthetic values were found lowest due to low PPFD and *gs*. Water and fertilization did affect the trend in the diurnal variation of *P*_*N*_. Under different soil moisture levels such as 100–95, 80–75, 55–50 and 35–30%, the *P*_*N*_ in the 300 mg L^−1^ treatment was higher than that of 500 mg L^−1^ and control (Figs. 3A–3P). The exogenous application of Si effects under non-limiting water and stressed conditions were significantly (*P* < 0.05) different at each measurement points (Table 1).

**Table 1:** Statistical analysis variance of four silicon concentrations on photosynthetic CO_2_ assimilation rate (*P*_N_), stomatal conductance (*gs*) and leaf transpirational rate (*E*) of *Saccharum officinarum* “GT 42” plants at diurnal and different soil water availabilities.

Treatment (%FC)	Time (h)					
	07:00	09:00	11:00	13:00	15:00	17:00
Effect of Si on *P*_N_						
100–95	NS	**	**	**	NS	NS
80–75	**	**	**	**	**	NS
55–50	**	NS	NS	NS	NS	NS
35–30	NS	NS	NS	NS	NS	NS
Effect of Si on *E*						
100–95	**	**	**	**	NS	**
80–75	**	**	**	**	NS	NS
55–50	**	**	**	**	**	NS
35–30	**	**	**	NS	NS	**
Effect of Si on gs						
100–95	**	**	NS	**	**	**
80–75	NS	**	**	**	NS	NS
55–50	NS	NS	NS	NS	NS	**
35–30	NS	NS	**	NS	**	NS

**Notes:**

The *Saccharum officinarum* plants were exposed to four soil water conditioNS (control, mild, moderate and severe drought, corresponding to available soil water capacity between 100–95%, 80–75%, 55–50% and 35–30% of the field capacity) and four levels of silicon (0: no Si, 100, 300 and 500 mg L^−1^, *n* = 5 for each treatment).

** Significant variatioNS between Si applicatioNS at specific time of the day for particular soil water field capacity (ANOVA, *P* < 0.05).

NS, no significant difference.

**Figure 4:**
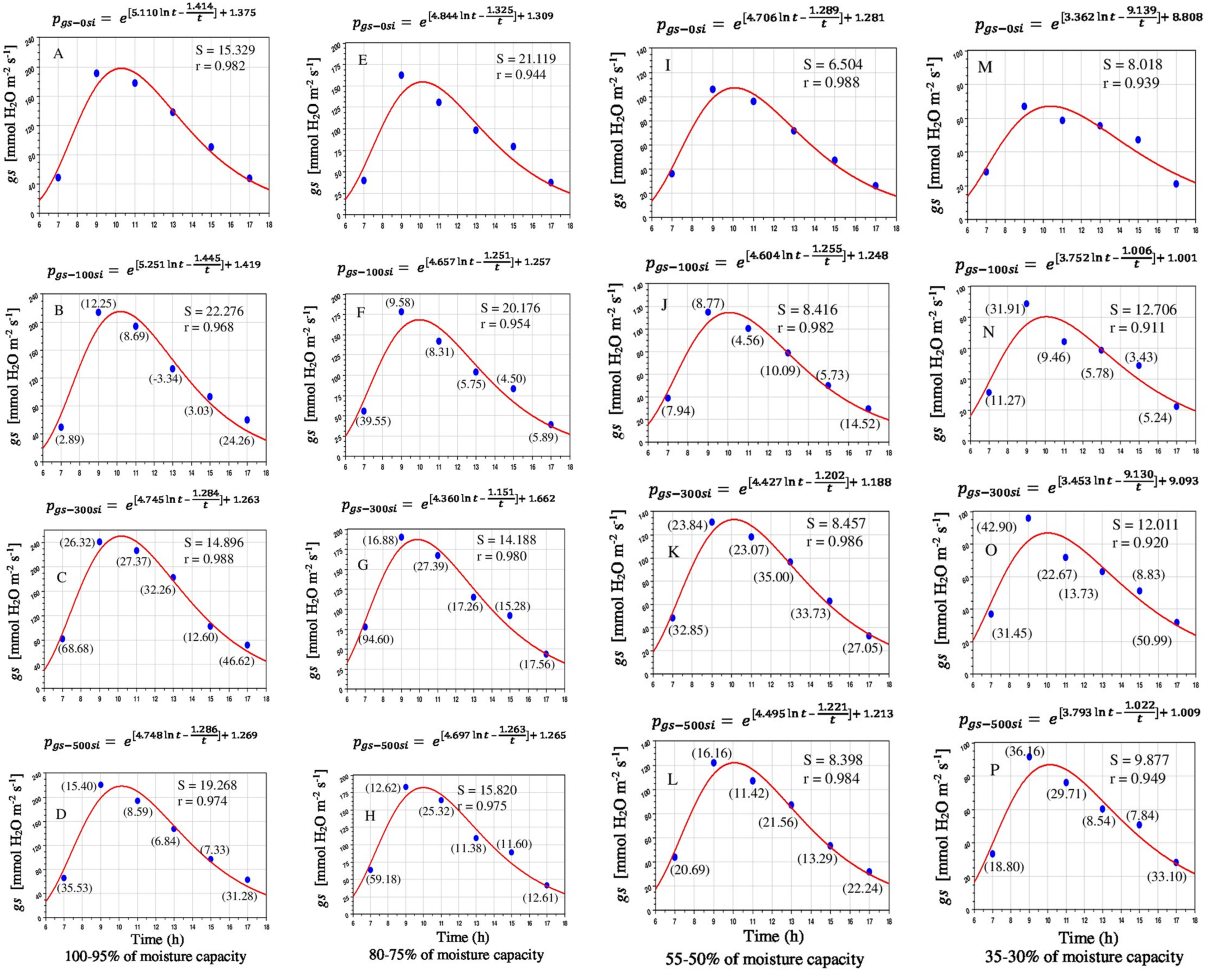
Diurnal variation of silicon on stomatal conductance in *Saccharum officinarm* “GT 42” plants under limited water supply such as 100–95% (A–D), 80–75% (E–H), 55–50% (I–L) and 35–30% of soil moisture capacity (M–P) with four levels of Si concentrations, for example, 0, 100, 300 and 500 mg L^−1^. Parenthesis values indicate percentage gain against control condition.

Stomata are the main limiting factor for carbon dioxide and vapor water exchange between plant leaves and the atmospheric conditions thus *gs* was directly controlled by *P*_*N*_ and *E*. The diurnal changes of *gs* under all water limitations and Si treatments showed similar trends (Fig. 3). The relatively high *gs* levels were noted at 9:00–10:00, due to compensation for transpirational water loss before dark. Then, *gs* was significantly declined until evening (11:00–17:00). The *gs* was decreased with reducing soil moisture levels. The *gs* of plants receiving the 300 mg L^−1^ was consistently up-regulated compared to that in the 500 mg L^−1^ Si and that without Si application (Figs. 4A–4P). The impacts of Si application on *gs* under control and water stressed plants were statistically significant (*P* < 0.05) (Table 1).

**Figure 5:**
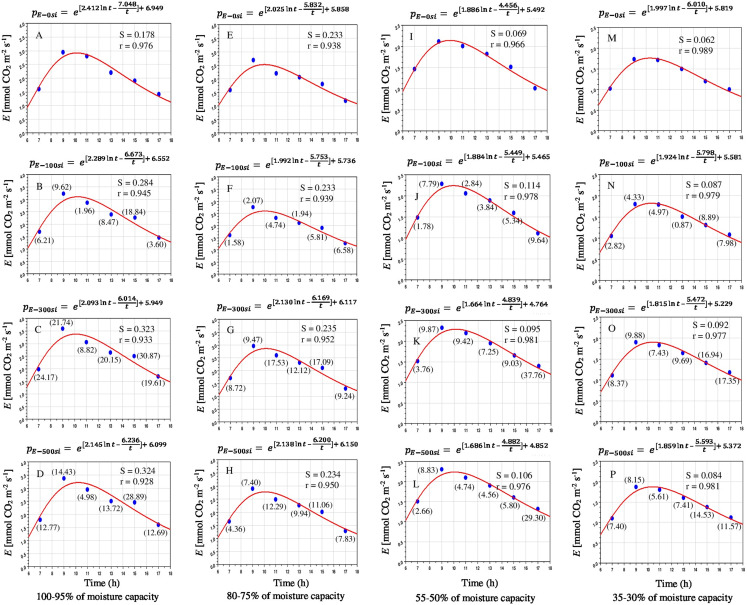
Diurnal variation in transpiration rate (100–95%, A–D; 80–75%, E–H; 55–50%, I–L and 35–30% of FC, M–P) of *Saccharum officinarm* “GT 42” plants exposed to four irrigation levels and four silicon levels, *n* = 5. Si = 0, 100, 300 and 500 mg L^−1^. Values in parentheses indicate percentage gain against control condition.

Leaf *E* water loss was compensated at dusk per day. Hence, based on the diurnal changes of the main environmental variables affecting leaf *E* under limited and sufficient soil moisture levels (Figs. 5A–5P). Under well and limited irrigation, leaf *E* was increased at a relatively higher from 7:00 to 9:00, followed by a significant and continuous decline from 11:00 to 17:00, reaching the minimum level at 17:00 (Fig. 5). Irrespective of Si application, *E* decreased with decreasing soil moisture capacity at each observation point during the day. Amended Si significantly affected leaf *E* at 100–95%, 80–75%, 55–50% and 35–30% of available soil moisture (Table 1). The exogenous application of Si enhanced the leaf *E* for most of the day time, especially in the plants grown under limited water irrigation.

It may be seen from Figs. 3 to 5 that *P*_N_, *gs* and *E* started increasing after the sun rise and reaches maximum between 9:00 and 10:00 and started declining thereafter. The model values of *P*_*N*_, *gs* and *E* matches well with the observed values of diurnal variations of photosynthetic responses under water stress and Si application. The *P*_*N*_ under mild, moderate and severe stress conditions gradually increased with increasing levels of Si. The correlation coefficients (*r*) between *P*_*N*_ and water stress levels were found 0.987, 0.966, 0.978, 0.974 and 0.990–0.995, 0.956–0.966, 0.972–0.982, 0.968–0.973, respectively, in the treatments applied Si. Similarly diurnal variations of *E* were well explained by the model with value of *r* as 0.938–0.989 without Si and 0.928–0.981 with Si under limited water. Diurnal variation of *g*_*s*_ was also well explained by the model with *r* as 0.939–0.988 without Si and 0.911–0.988 under limited water with Si. Limited water supply drastically suppressed the photosynthetic responses of *Saccharum officinarum* plant leaves (Table S1).

## Discussion

Sufficient water is important for the appropriate growth and development of plants. Its effect on plants depends on their developmental stage. Water stress is one of the major environmental factors that affect the plant development, cellular, metabolism, productivity and quality of *Saccharum officinarum* (Verma et al., 2019a, 2019b, 2020). Higher plants have developed different types of mechanisms to tolerate stresses. The protective roles of Si in combating various environmental stresses have been widely reported (Van Bockhaven, De Vleesschauwer & Höfte, 2013; Zhu & Gong, 2014; Verma et al., 2019a, 2020). In this study, the protective role of Si was investigated in *Saccharum officinarum* plants during insufficient water supply.

In our study, the growth and biomass of *Saccharum officinarum* plants were markedly down-regulated after the plants were subjected to stress. However, the application of Si with soil irrigation decreased the severity of water stressed growth inhibition. It enhanced *Saccharum officinarum* tolerance to water stress in terms of promoting the plant’s growth and development (Fig. 2). Previous research has shown that Si application could increase water stress tolerance of plants. However, most of the studies have been conducted on Si-accumulating plants, whereas less information is available regarding the role of Si on water deficit tolerance of plants (Nikolic et al., 2007). The increase in growth and biomass accumulation is endorsed to the higher *P*_*N*_ due to improved photosynthetic capacity of the stressed plants, which is in agreed with the previous demonstrations (Zhu & Gong, 2014; Liu et al., 2014; Shi et al., 2016; Verma et al., 2019a, 2020).

Our results further confirmed the findings of the previous reports that application of Si mitigated water stress and significantly affected plant growth and development in *Oryza sativa* (Ming et al., 2012), *Zea mays* (Kaya, Tuna & Higgs, 2006), *Triticum aestivum* (Pei et al., 2010; Gong & Chen, 2012), *Sorghum bicolor* (Liu et al., 2014), *Solanum lycopersicum* (Shi et al., 2016) and *Saccharum officinarum* (Verma et al., 2019a, 2020). Our results also imply a potential application of Si fertilizer in *Saccharum officinarum* crop production in tropical and sub-tropical regions.

In accordance with our experimental findings, leaf gas exchange significantly decreased during water stress with increasing stress levels (Figs. 3–5). Water deficit causes a major loss in LAE, LWC and photosynthetic pigments, which undeniably impairs and decreases *P*_*N*_, directly affecting plant performance (Shi et al., 2016; Verma et al., 2019a; Liang et al., 2019). Si-mediated up-regulation of leaf *P*_*N*_ under drought could be attributed to improved or upgraded plant water status. In this experiment, the water status of *Saccharum officinarum* leaves were significantly improved by applied Si during drought. Our results showed that the applied Si mitigated the negative effects of water stress and enhanced the growth and biomass, improved the photosynthetic performance compared with the control plants during stress condition (Figs. 2–5). Our observations regarding enhancement in plant development and photosynthetic parameters due to exogenous use of Si during water stress condition are in accordance with other observations in various crops (Hattori et al., 2005; Pei et al., 2010; Ming et al., 2012; Shi et al., 2016; Chen et al., 2018; Ali et al., 2018; Verma et al., 2020). The improvement of growth by application of Si corresponded to the maintenance of higher *P*_*N*_ (Fig. 3).

Drought stress can severely decrease the yield of *Saccharum officinarum* by inhibiting the photosynthetic responses (Passioura, 2007; Santos et al., 2009; Verma et al., 2019b, 2019c). At the initial sign of drought stress, plants close the stomata to avoid excess water loss by *E* and as a consequence, under moderate stress condition, *P*_*N*_ is affected and is eventually inhibited by enhancing stress severity (Verma et al., 2019a, 2020). However, previous findings stated that an optimum level of Si fertilizer enhanced/improved the stomatal mechanisms/functions by enabling plants to reopen their stomata during water-deficit (Gong et al., 2005; Kaya, Tuna & Higgs, 2006; Chen et al., 2011; Liu et al., 2014; Verma et al., 2020). In this study, *P*_*N*_ was significantly low in the water-stressed plants without Si application, undoubtedly due to stomatal limitations. Si treatments, however, were found to escalate *P*_*N*_ and *gs*, and cause a simultaneous enhancement in LWC. The moderate to higher concentration of Si (300–500 mg L^−1^) as soil irrigation were more effective in enhancing growth and photosynthetic performance during water stress condition (Figs. 2–5).

In this study, however, the *E* of *Saccharum officinarum* leaves was enhanced by applied Si under drought stress (Fig. 5). The supplied Si treatments also resulted in an enhancement of *E*, possibly driven by the increased *gs* to maintain a steady state of *P*_*N*_ against stress. Our results are in accordance with the previous studies like wheat, sorghum, rice, maize and tomato (Gong et al., 2005; Hattori et al., 2005; Chen et al., 2011; Shi et al., 2016) during abiotic stresses. Water deficiency and Si fertilization slightly affected the diurnal variations of *Saccharum officinarum* plants, which are positively correlated to the biological rhythm of the plants (Chen et al., 2016). However, compared with the plants cultivated under limited water without Si, Si generally enhanced plant growth and photosynthetic responses. An increase in soil moisture capacity is more effective than an increase in nutrient supply in improving the plant growth and development of *Saccharum officinarum* plants. Improvement of photosynthesis is the most important factor for overall plant performance. The change of PPFD during the natural diurnal cycle is the most important factor driving photosynthetic parameters (Paul & Pellny, 2003). Thus with maximum air RH and fully-irrigated plants, gas exchange changes in *Saccharum officinarum* plants is mainly driven by variations in sunlight intensity (Du et al., 2000), mainly resulting from the significantly direct or indirect dependance of C_4_ photosynthetic enzymes to light intensity (Leegood & Walker, 1999; De Souza et al., 2018; Verma et al., 2019a). Stomata-related increment of *P*_*N*_ was found when Si improved *gs* and *E* (Chen et al., 2011; Savvas & Ntatsi, 2015; Verma et al., 2019b, 2019c).

The proposed model fitted quite well with the observed *P*_*N*_, *gs* and *E*. The *P*_*N*_ fitted with “*r”* ranging from 0.956 to 0.995 and “S” 1.080 to 2.431; *gs* with “*r*” ranging from 0.911 to 0.988 and “S” from 6.540 to 22.276 and *E* with “*r*” from 0.928 to 0.989 and “S” from 0.062 to 0.324, respectively. The variations of model constants have a consistency in variations with changes in moisture status and applied Si levels (Figs. 3–5). The photosynthetic responses could be obtained for intermediate value of Si and moisture content of the soil by selecting the appropriate values of model constants. The model could be used to assess the photosynthetic advantages of Si application against water stress by integrating the model in terms of numerical values.

In summary, the results of this study revealed that application of Si might be an efficient approach for enhancing tolerance of *Saccharum officinarum* plants against water stress. It is also increased the growth, biomass accumulation and photosynthesis by protecting the leaf chlorophyl from degradation in *Saccharum officinarum* plants during stress. Further studies are needed to explore, how Si triggers the photosynthetic defense mechanism in *Saccharum officinarum* plants during drought stress. Thus, appropriate concentration is recommended for various crops to mitigate abiotic stresses.

## Supplemental Information

10.7717/peerj.10154/supp-1 Supplemental Information 1 Diurnal variation of cumulative responses of leaf gas exchange. Model constant and cumulative diurnal variation of photosynthetic parameters of limited water supply with different silicon levels.

10.7717/peerj.10154/supp-2 Supplemental Information 2 Experimental raw data.

10.7717/peerj.10154/supp-3 Supplemental Information 3 Model constants and cumulative photosynthetic responses.

## Additional Information and Declarations

### Data Availability

The following information was supplied regarding data availability: Raw measurements are available as a Supplemental File.
