# Supplementary material for: Developing mathematical model for diurnal dynamics of photosynthesis in Saccharum officinarum responsive to different irrigation and silicon application
Source: PeerJ. 2020 Oct 27;8:e10154. doi: 10.7717/peerj.10154 (PMC7597626; doi:10.7717/peerj.10154)
Supplement: Supplemental Information 1 — Model constant and cumulative diurnal variation of photosynthetic parameters of limited water supply with different silicon levels. [file peerj-08-10154-s001.pdf]

Manuscript title

Developing mathematical model for diurnal dynamics of photosynthesis in *Saccharum officinarum* responsive to different irrigation and silicon application

Name(s) of the author(s)

Krishan K. Verma[1], Kai-Chao Wu[1], Chhedi Lal Verma[2], Dong-Mei Li[1], Mukesh Kumar Malviya[1], Rajesh Kumar Singh[1], Pratiksha Singh[1], Gan-Lin Chen[3], Xiu-Peng Song[1,*], Yang-Rui Li[1,*]

Affiliation(s) and address(es) of the author(s)

[1]Key Laboratory of Sugarcane Biotechnology and Genetic Improvement (Guangxi), Ministry of Agriculture and Rural Affairs/ Guangxi Key Laboratory of Sugarcane Genetic Improvement/ Sugarcane Research Institute, Guangxi Academy of Agricultural Sciences, Nanning - 530 007, China; drvermakishan@gmail.com; ORCID – 0000-0002-5501-7905 (KKV), kaichaowu@126.com (K.C.W), Domi_li@163.com (D.M.L.), rajeshsingh999@gmail.com (R.K.S.), singh.pratiksha23@gmail.com (P.S.), mkshmalviya@yahoo.com (M.K.M.)

[2]Central Soil Salinity Research Institute (RRS), Lucknow – 226 005, India; lalc_verma@yahoo.com

[3]Institute of Biotechnology, Guangxi Academy of Agricultural Sciences, Nanning - 530 007, China; ganlin-chen@163.com (G.L.C.)

*Corresponding: xiupengsong@163.com (X.P.S.); liyr@gxaas.net (Y.R.L.);

Tel.: +86-18677128565 (X.P.S); 13807883389 (Y.R.L.);

ORCID: 0000-0002-7559-9244 (Y.R.L.)

Supplementary file

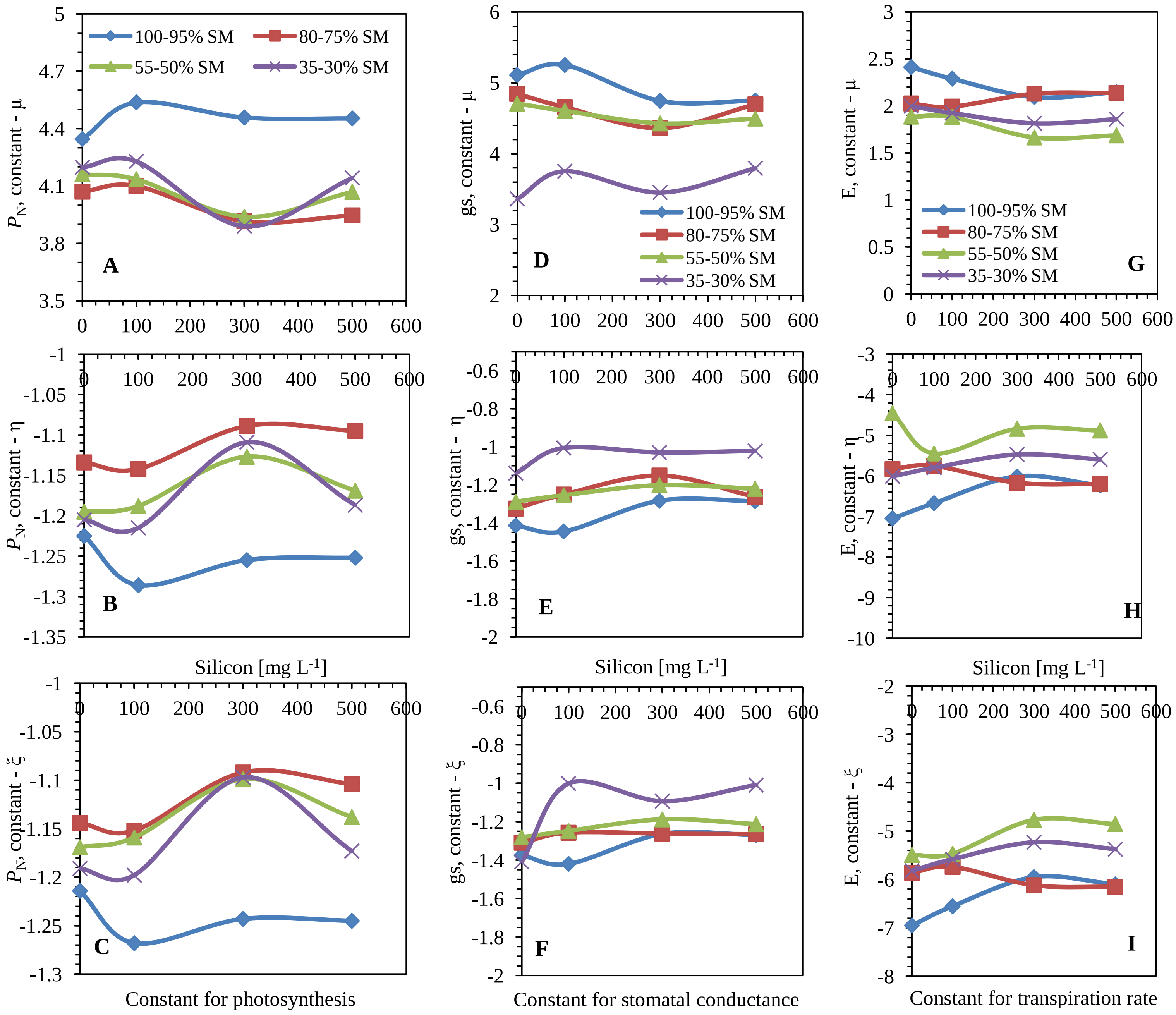

Fig. 1S: Model constants for photosynthesis (A-C), stomatal conductance (D-F) and transpiration rate (G-I).

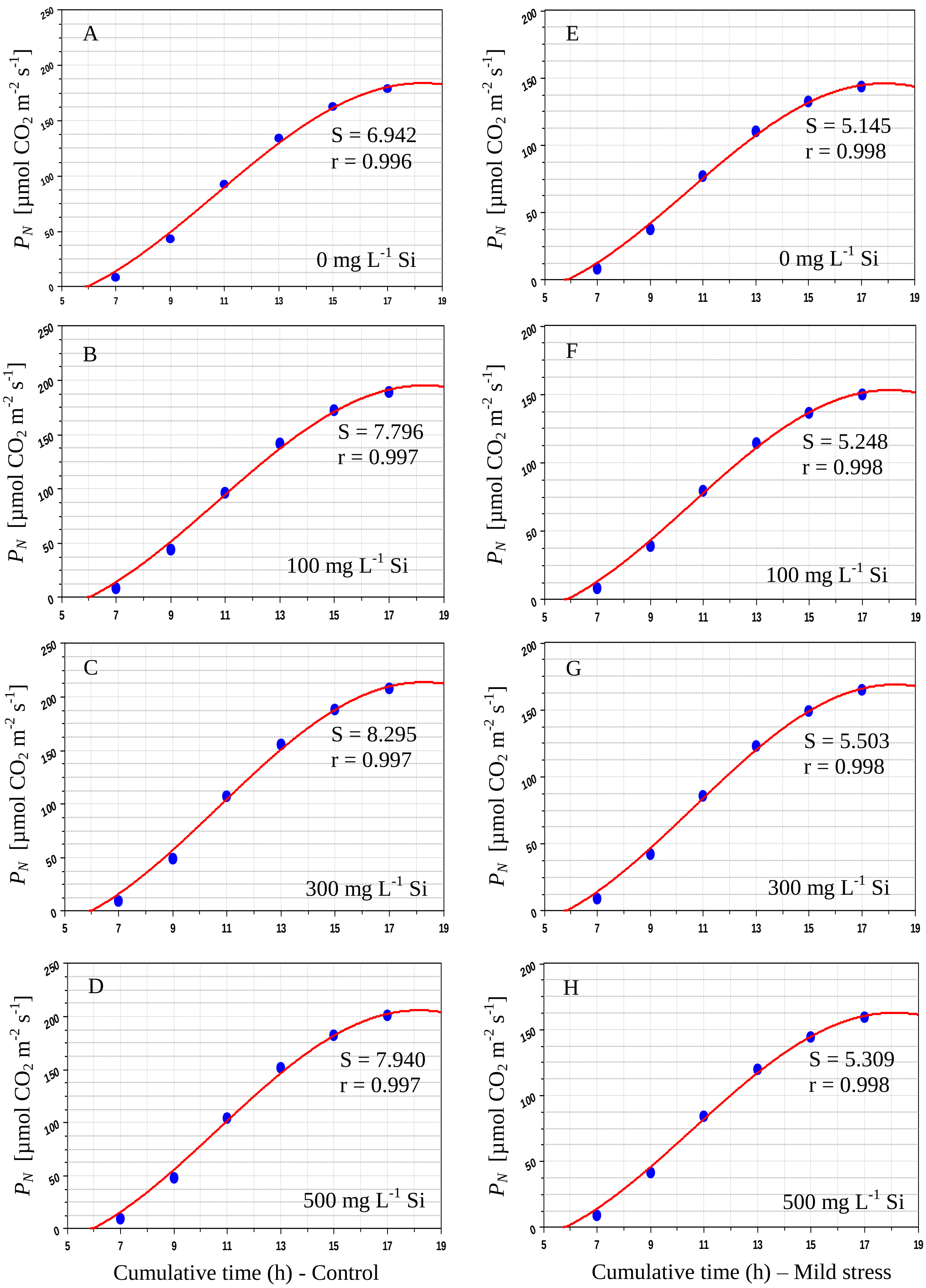
A
S = 6.942
r = 0.996
0 mg L-1 Si
B
S = 7.796
r = 0.997
100 mg L-1 Si
C
S = 8.295
r = 0.997
300 mg L-1 Si
D
S = 7.940
r = 0.997
500 mg L-1 Si
E
S = 5.145
r = 0.998
0 mg L-1 Si
F
S = 5.248
r = 0.998
100 mg L-1 Si
G
S = 5.503
r = 0.998
300 mg L-1 Si
H
S = 5.309
r = 0.998
500 mg L-1 Si
PN [µmol CO2 m-2 s-1]
Cumulative time (h) - Control
Cumulative time (h) – Mild stress

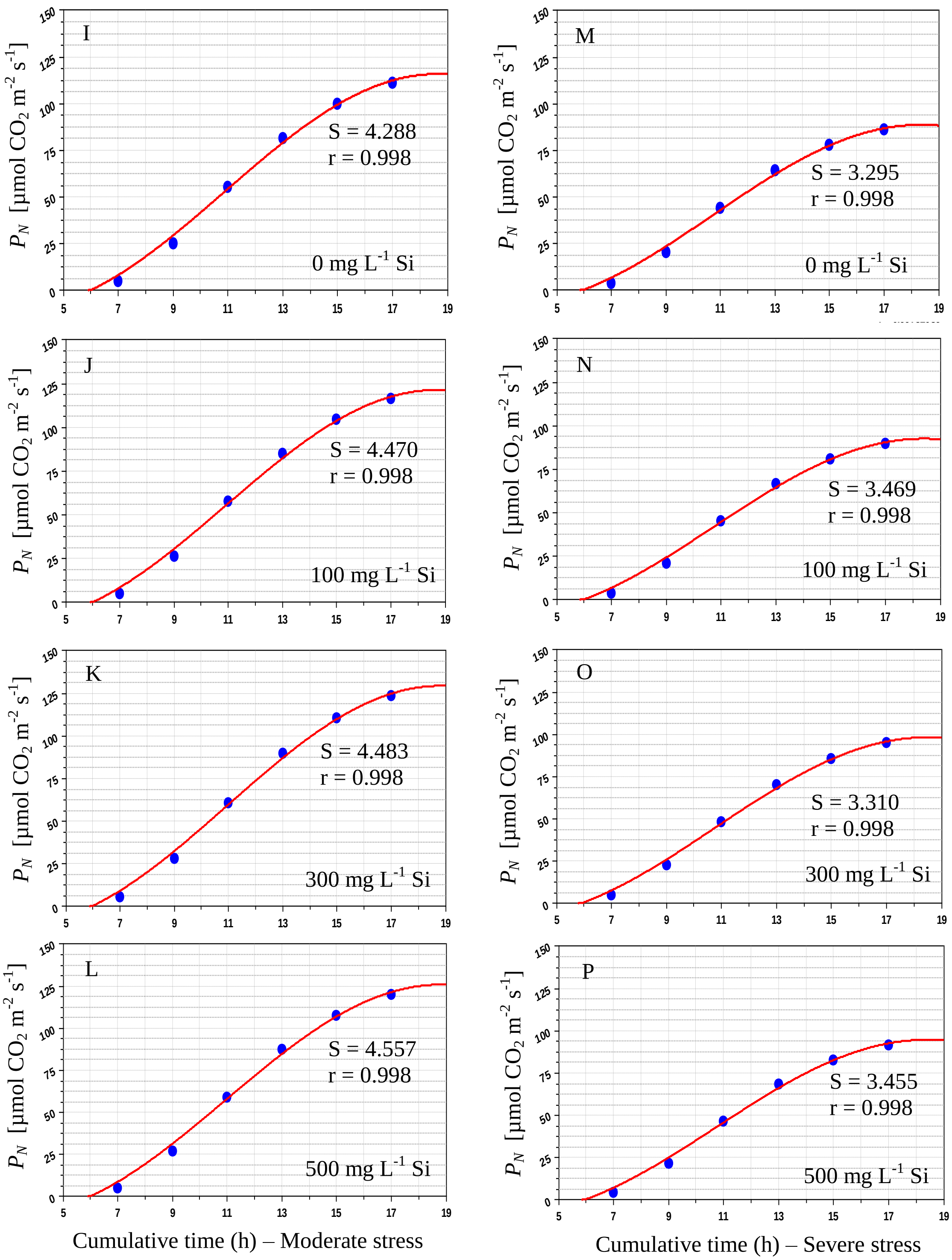

Fig. 2S: Cumulative diurnal variation of photosynthesis (P_N, µmol CO_2 $m^{-2}s^{-1}$) in *Saccharum officinarum* plant leaves under control (A-D) and limited water supply [mild (E-H), moderate (I-L), and severe stress (M-P)] with different silicon levels (0, 100, 300 and 500 mg L^{-1}) application. S = standard error, r = correlation coefficient.

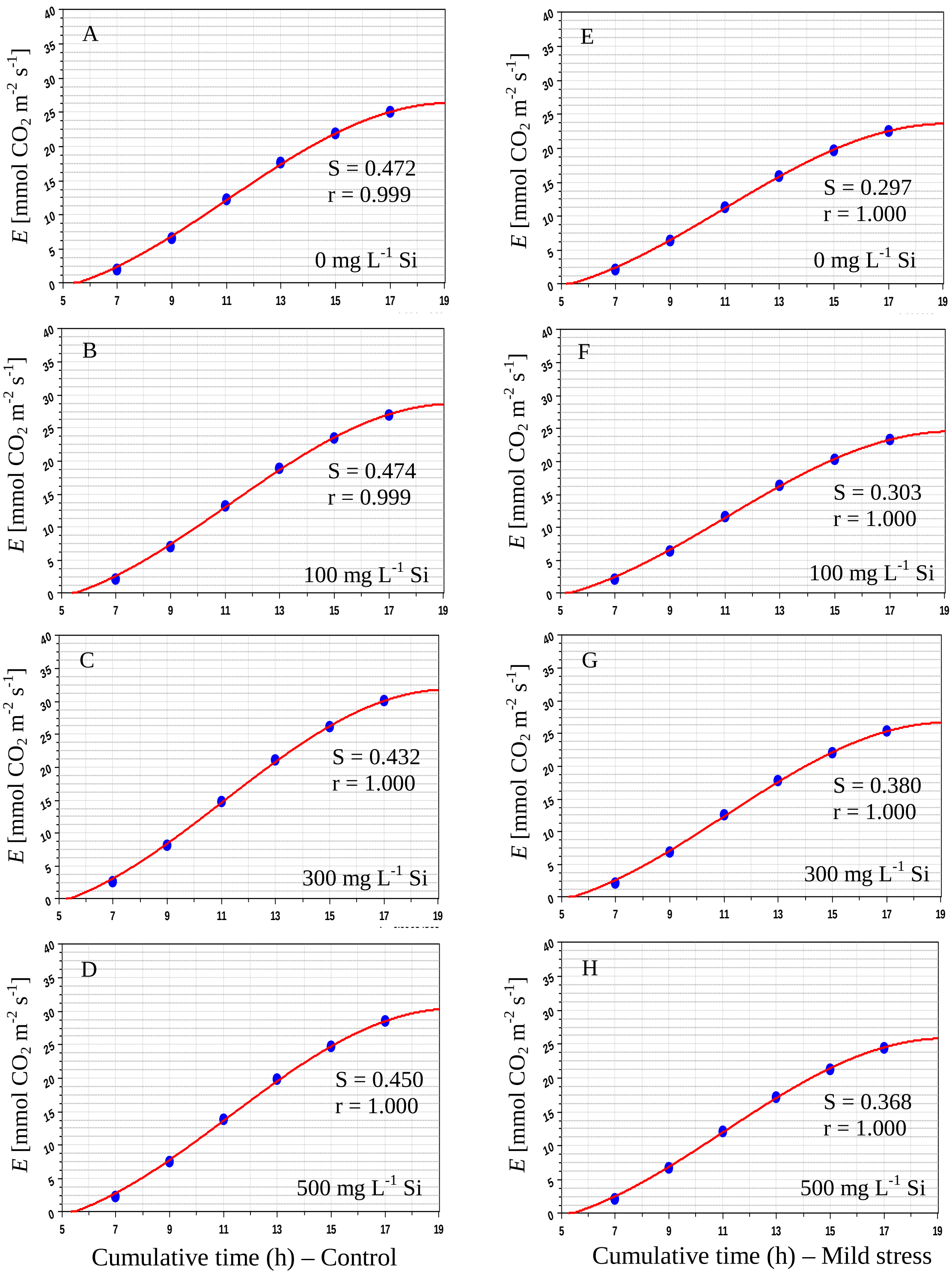
A
S = 0.472
r = 0.999
0 mg L-1 Si
B
S = 0.474
r = 0.999
100 mg L-1 Si
C
S = 0.432
r = 1.000
300 mg L-1 Si
D
S = 0.450
r = 1.000
500 mg L-1 Si
E
S = 0.297
r = 1.000
0 mg L-1 Si
F
S = 0.303
r = 1.000
100 mg L-1 Si
G
S = 0.380
r = 1.000
300 mg L-1 Si
H
S = 0.368
r = 1.000
500 mg L-1 Si
E [mmol CO2 m-2 s-1]
Cumulative time (h) – Control
Cumulative time (h) – Mild stress

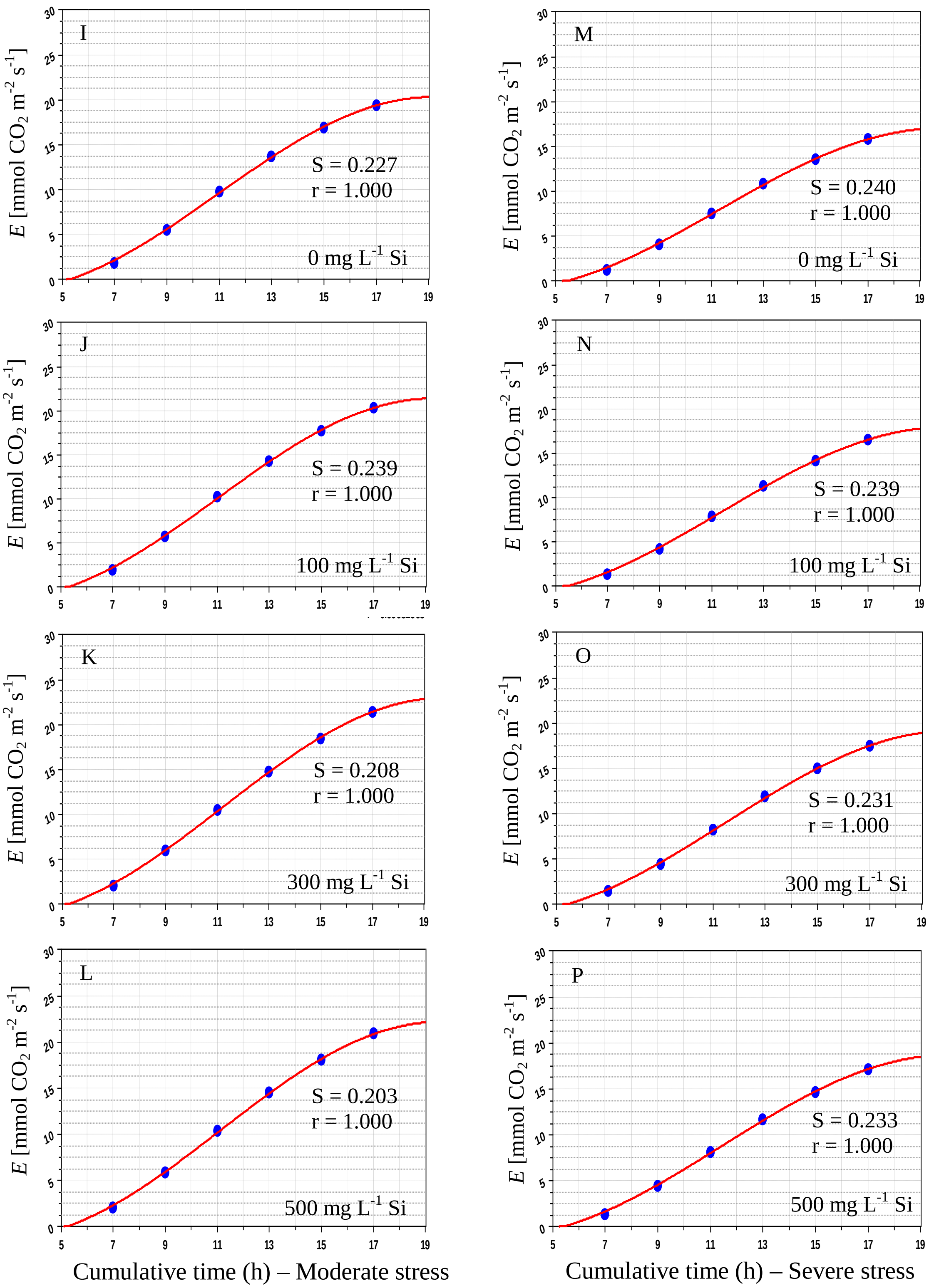

Fig. 3S: Cumulative diurnal variations of transpiration rate (*E*, mmol CO_2 $m^{-2}s^{-1}$) in *Saccharum officinarum* plant leaves under control (A-D) and limited water supply [mild (E-H), moderate (I-L), and severe stress (M-P)] with different levels of silicon (0, 100, 300 and 500 mg L^{-1}) application. S = standard error, r = correlation coefficient.

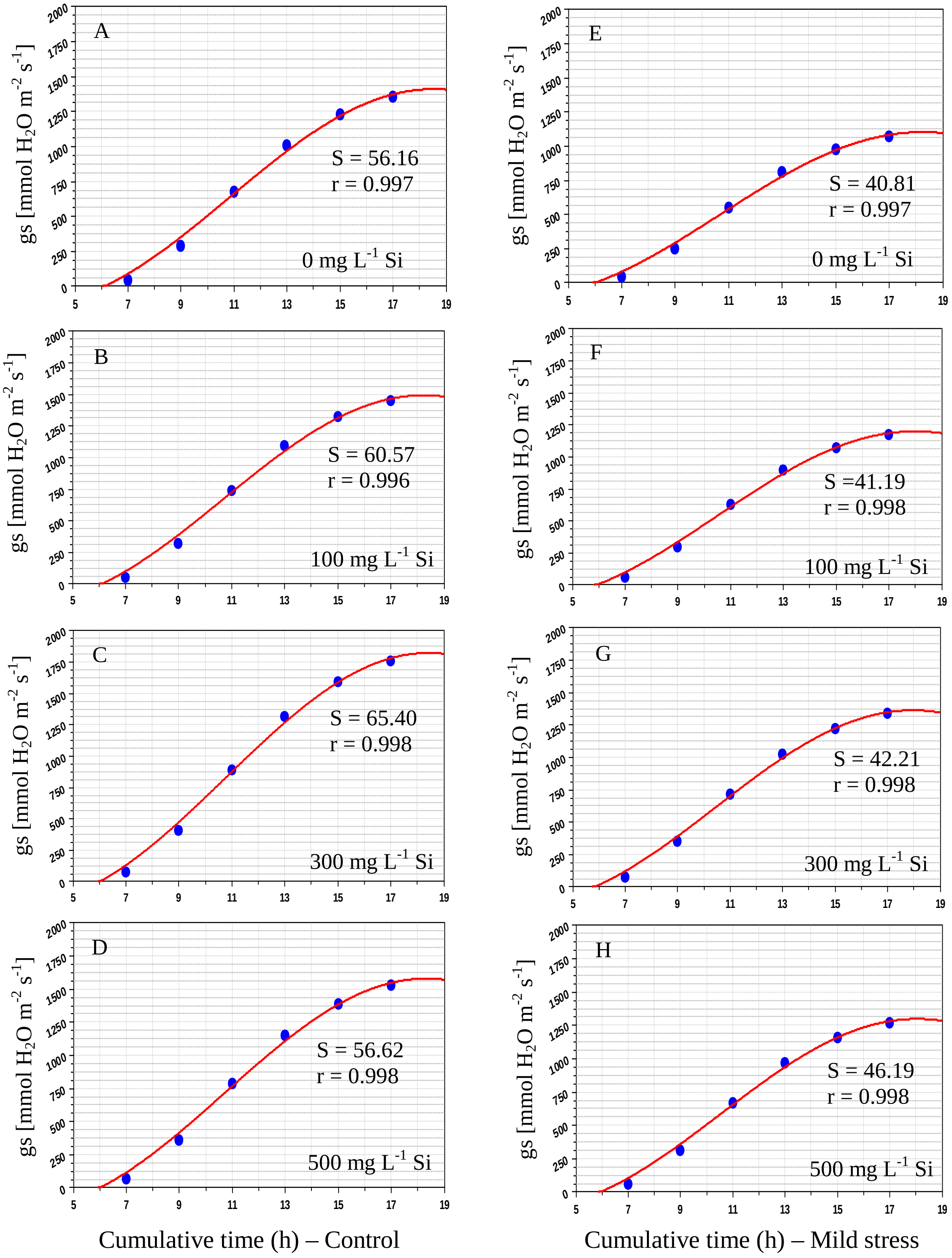
gs [mmol H_2O m^{-2} s^{-1}]
A
S = 56.16
r = 0.997
0 mg L^{-1} Si
B
S = 60.57
r = 0.996
100 mg L^{-1} Si
C
S = 65.40
r = 0.998
300 mg L^{-1} Si
D
S = 56.62
r = 0.998
500 mg L^{-1} Si
Cumulative time (h) – Control
E
S = 40.81
r = 0.997
0 mg L^{-1} Si
F
S =41.19
r = 0.998
100 mg L^{-1} Si
G
S = 42.21
r = 0.998
300 mg L^{-1} Si
H
S = 46.19
r = 0.998
500 mg L^{-1} Si
Cumulative time (h) – Mild stress

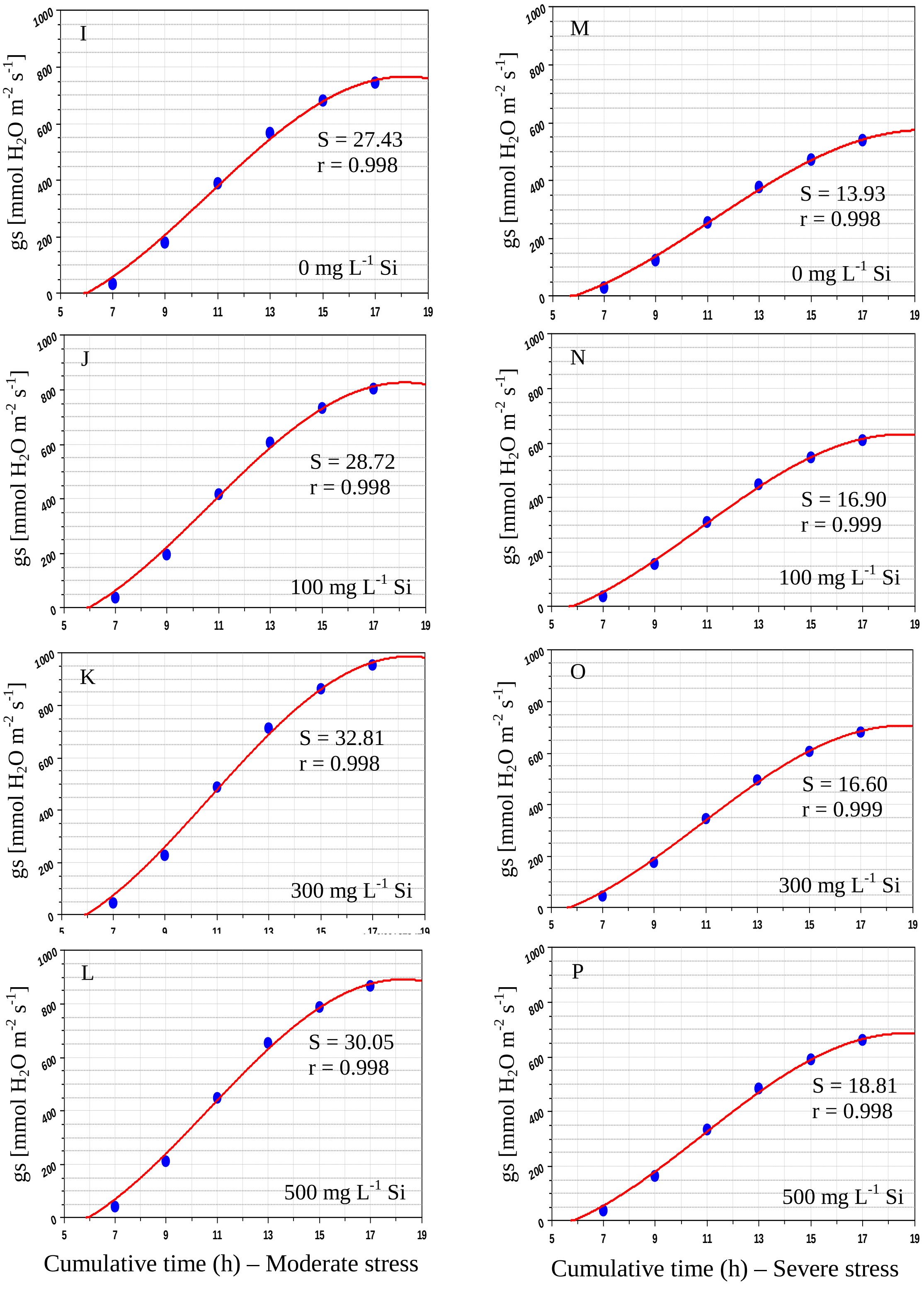

Fig. 4S: Cumulative diurnal variations of stomatal conductance (gs, mmol H_2O $m^{-2}s^{-1}$) in *Saccharum officinarum* plant leaves under control (A-D) and limited water supply [mild (E-H), moderate (I-L), and severe stress (M-P)] with different levels of silicon (0, 100, 300 and 500 mg L^{-1}) application. S = standard error, r = correlation coefficient.
