# Supplementary material for: Developing mathematical model for diurnal dynamics of photosynthesis in Saccharum officinarum responsive to different irrigation and silicon application
Source: PeerJ. 2020 Oct 27;8:e10154. doi: 10.7717/peerj.10154 (PMC7597626; doi:10.7717/peerj.10154)
Supplement: Supplemental Information 3 [file peerj-08-10154-s003.docx]

**Manuscript title**

Developing mathematical model for diurnal dynamics of photosynthesis in *Saccharum officinarum* responsive to different irrigation and silicon application

**Name(s) of the author(s)**

Krishan K. Verma^1^, Kai-Chao Wu^1^, Chhedi Lal Verma^2^, Dong-Mei Li^1^, Mukesh Kumar Malviya^1^, Rajesh Kumar Singh^1^, Pratiksha Singh^1^, Gan-Lin Chen^3^, Xiu-Peng Song^1,*^, Yang-Rui Li^1,*^

**Affiliation(s) and address(es) of the author(s)**

^1^Key Laboratory of Sugarcane Biotechnology and Genetic Improvement (Guangxi), Ministry of Agriculture and Rural Affairs/ Guangxi Key Laboratory of Sugarcane Genetic Improvement/ Sugarcane Research Institute, Guangxi Academy of Agricultural Sciences, Nanning - 530 007, China; [drvermakishan@gmail.com](mailto:drvermakishan@gmail.com); ORCID – 0000-0002-5501-7905 (KKV), [kaichaowu@126.com](mailto:kaichaowu@126.com) (K.C.W), [Domi_li@163.com](mailto:Domi_li@163.com) (D.M.L.), [rajeshsingh999@gmail.com](mailto:rajeshsingh999@gmail.com) (R.K.S.), [singh.pratiksha23@gmail.com](mailto:singh.pratiksha23@gmail.com) (P.S.), [mkshmalviya@yahoo.com](mailto:mkshmalviya@yahoo.com) (M.K.M.)

^2^Central Soil Salinity Research Institute (RRS), Lucknow – 226 005, India; [lalc_verma@yahoo.com](mailto:lalc_verma@yahoo.com)

^3^Institute of Biotechnology, Guangxi Academy of Agricultural Sciences, Nanning - 530 007, China; [ganlin-chen@163.com](mailto:ganlin-chen@163.com) (G.L.C.)

*Corresponding: [xiupengsong@163.com](mailto:xiupengsong@163.com) (X.P.S.); [liyr@gxaas.net](mailto:liyr@gxaas.net) (Y.R.L.); Tel.: +86-18677128565 (X.P.S); 13807883389 (Y.R.L.);

ORCID: 0000-0002-7559-9244 (Y.R.L.)

**Table 1S:** Model constants for photosynthetic responses.

| Photosynthetic responses | Soil moisture (%] | Silicon  [mg L^-1^] | µ | ɳ | ξ | r | S |
| --- | --- | --- | --- | --- | --- | --- | --- |
| Photosynthesis  [µmol CO_2_ m^-2^ s^-1^] | 100-95 | 0 | 4.345 | -1.225 | -1.214 | 0.987 | 1.548 |
|  |  | 100 | 4.537 | -1.286 | -1.268 | 0.990 | 1.453 |
|  |  | 300 | 4.458 | -1.255 | -1.243 | 0.995 | 1.080 |
|  |  | 500 | 4.453 | -1.252 | -1.245 | 0.992 | 1.407 |
|  | 80-75 | 0 | 4.070 | -1.134 | -1.144 | 0.966 | 1.981 |
|  |  | 100 | 4.100 | -1.142 | -1.152 | 0.962 | 2.186 |
|  |  | 300 | 3.916 | -1.089 | -1.092 | 0.965 | 2.195 |
|  |  | 500 | 3.946 | -1.095 | -1.104 | 0.956 | 2.431 |
|  | 55-50 | 0 | 4.161 | -1.195 | -1.169 | 0.978 | 1.215 |
|  |  | 100 | 4.135 | -1.188 | -1.159 | 0.972 | 1.438 |
|  |  | 300 | 3.939 | -1.127 | -1.099 | 0.982 | 1.148 |
|  |  | 500 | 4.069 | -1.169 | -1.138 | 0.978 | 1.284 |
|  | 35-30 | 0 | 4.197 | -1.205 | -1.191 | 0.974 | 1.056 |
|  |  | 100 | 4.228 | -1.215 | -1.198 | 0.973 | 1.118 |
|  |  | 300 | 3.890 | -1.109 | -1.097 | 0.968 | 1.204 |
|  |  | 500 | 4.142 | -1.187 | -1.173 | 0.969 | 1.192 |
| Stomatal conductance  [mmol H_2_O m^-2^ s^-1^] | 100-95 | 0 | 5.110 | -1.414 | -1.375 | 0.982 | 15.329 |
|  |  | 100 | 5.251 | -1.445 | -1.419 | 0.968 | 22.276 |
|  |  | 300 | 4.745 | -1.284 | -1.263 | 0.988 | 14.896 |
|  |  | 500 | 4.748 | -1.286 | -1.269 | 0.974 | 19.268 |
|  | 80-75 | 0 | 4.844 | -1.325 | -1.309 | 0.944 | 21.119 |
|  |  | 100 | 4.657 | -1.251 | -1.257 | 0.954 | 20.176 |
|  |  | 300 | 4.360 | -1.151 | -1.662 | 0.980 | 14.188 |
|  |  | 500 | 4.697 | -1.263 | -1.265 | 0.975 | 15.820 |
|  | 55-50 | 0 | 4.706 | -1.289 | -1.281 | 0.988 | 6.504 |
|  |  | 100 | 4.604 | -1.255 | -1.248 | 0.982 | 8.416 |
|  |  | 300 | 4.427 | -1.202 | -1.188 | 0.986 | 8.457 |
|  |  | 500 | 4.495 | -1.221 | -1.213 | 0.984 | 8.398 |
|  | 35-30 | 0 | 3.362 | -1.139 | -1.408 | 0.939 | 8.018 |
|  |  | 100 | 3.752 | -1.006 | -1.001 | 0.911 | 12.706 |
|  |  | 300 | 3.453 | -1.030 | -1.093 | 0.920 | 12.011 |
|  |  | 500 | 3.793 | -1.022 | -1.009 | 0.949 | 9.877 |
| Transpiration rate  [mmol CO_2_ m^-2^ s^-1^] | 100-95 | 0 | 2.412 | -7.048 | -6.949 | 0.976 | 0.178 |
|  |  | 100 | 2.289 | -6.673 | -6.552 | 0.945 | 0.284 |
|  |  | 300 | 2.093 | -6.014 | -5.950 | 0.933 | 0.323 |
|  |  | 500 | 2.145 | -6.236 | -6.099 | 0.928 | 0.324 |
|  | 80-75 | 0 | 2.025 | -5.832 | -5.858 | 0.938 | 0.233 |
|  |  | 100 | 1.992 | -5.753 | -5.736 | 0.939 | 0.233 |
|  |  | 300 | 2.130 | -6.169 | -6.117 | 0.952 | 0.235 |
|  |  | 500 | 2.138 | -6.200 | -6.150 | 0.950 | 0.234 |
|  | 55-50 | 0 | 1.886 | -4.456 | -5.492 | 0.966 | 0.069 |
|  |  | 100 | 1.884 | -5.449 | -5.465 | 0.978 | 0.114 |
|  |  | 300 | 1.664 | -4.839 | -4.764 | 0.981 | 0.095 |
|  |  | 500 | 1.686 | -4.882 | -4.852 | 0.976 | 0.106 |
|  | 35-30 | 0 | 1.997 | -6.010 | -5.819 | 0.989 | 0.062 |
|  |  | 100 | 1.924 | -5.798 | -5.581 | 0.979 | 0.087 |
|  |  | 300 | 1.815 | -5-472 | -5.229 | 0.977 | 0.092 |
|  |  | 500 | 1.859 | -5.593 | -5.372 | 0.981 | 0.084 |

**Table 2S:** Diurnal variation of cumulative photosynthetic rate in *Saccharum officinarum* ‘GT 42’ plants grown under different soil moisture capacity and silicon.

| Time  (h) | Photosynthesis (µmol CO_2_ m^-2^s^-1^), irrigation level (100-95% of FC) with Si (mg L^-1^) | | | | | | | |
| --- | --- | --- | --- | --- | --- | --- | --- | --- |
|  | Cumulative | Cumulative (%) | Cumulative | Cumulative (%) | Cumulative | Cumulative (%) | Cumulative | Cumulative (%) |
|  | 0 |  | 100 |  | 300 |  | 500 |  |
| 5:00 | 0.00 | - | 0.00 | - | 0.00 | - | 0.00 | - |
| 7:00 | 8.25 | 4.62 | 7.98 | 4.21 | 9.27 | 4.46 | 9.20 | 4.59 |
| 9:00 | 42.76 | 23.92 | 43.97 | 23.23 | 49.31 | 23.73 | 48.38 | 24.11 |
| 11:00 | 91.93 | 51.43 | 96.74 | 51.08 | 106.94 | 51.46 | 104.16 | 51.91 |
| 13:00 | 133.97 | 74.95 | 142.00 | 74.97 | 156.11 | 75.12 | 151.37 | 75.44 |
| 15:00 | 162.07 | 90.67 | 171.94 | 90.78 | 188.70 | 90.80 | 182.49 | 90.95 |
| 17:00 | 178.74 | 100.00 | 189.40 | 100.00 | 207.81 | 100.00 | 200.65 | 100.00 |
| Irrigation level (80-75% of FC) with Si (mg L^-1^) | | | | | | | | |
| 5:00 | 0.00 | - | 0.00 | - | 0.00 | - | 0.00 | - |
| 7:00 | 8.13 | 5.67 | 8.35 | 5.56 | 9.27 | 5.63 | 9.21 | 5.78 |
| 9:00 | 37.72 | 26.32 | 38.95 | 25.94 | 42.07 | 25.54 | 41.50 | 26.04 |
| 11:00 | 77.19 | 53.86 | 79.83 | 53.17 | 85.97 | 52.19 | 84.22 | 52.86 |
| 13:00 | 110.13 | 76.84 | 113.89 | 75.86 | 123.31 | 74.86 | 120.14 | 75.40 |
| 15:00 | 132.12 | 92.19 | 136.57 | 90.97 | 148.90 | 90.40 | 144.47 | 90.67 |
| 17:00 | 143.32 | 100.00 | 150.13 | 100.00 | 164.71 | 100.00 | 159.34 | 100.00 |
| Irrigation level (55-50% of FC) with Si (mg L^-1^) | | | | | | | | |
| 5:00 | 0.00 | - | 0.00 | - | 0.00 | - | 0.00 | - |
| 7:00 | 4.86 | 4.37 | 5.05 | 4.34 | 5.70 | 4.61 | 5.23 | 4.35 |
| 9:00 | 25.38 | 22.81 | 26.32 | 22.63 | 28.27 | 22.87 | 27.02 | 22.48 |
| 11:00 | 55.34 | 49.74 | 57.51 | 49.45 | 60.80 | 49.19 | 58.99 | 49.07 |
| 13:00 | 81.77 | 73.50 | 85.18 | 73.24 | 89.88 | 72.72 | 87.56 | 72.84 |
| 15:00 | 100.04 | 89.92 | 104.42 | 89.78 | 110.51 | 89.42 | 107.65 | 89.55 |
| 17:00 | 111.25 | 100.00 | 116.31 | 100.00 | 123.59 | 100.00 | 120.21 | 100.00 |
| Irrigation level (35-30% of FC) with Si (mg L^-1^) | | | | | | | | |
| 5:00 | 0.00 | - | 0.00 | - | 0.00 | - | 0.00 | - |
| 7:00 | 3.99 | 4.64 | 4.06 | 4.53 | 4.86 | 5.11 | 4.32 | 4.71 |
| 9:00 | 20.47 | 23.79 | 21.10 | 23.55 | 23.04 | 24.24 | 21.85 | 23.82 |
| 11:00 | 43.96 | 51.09 | 45.57 | 50.85 | 48.25 | 50.77 | 46.74 | 50.95 |
| 13:00 | 64.18 | 74.59 | 66.72 | 74.46 | 70.19 | 73.86 | 68.25 | 74.40 |
| 15:00 | 77.84 | 90.47 | 81.03 | 90.43 | 85.48 | 89.95 | 82.88 | 90.34 |
| 17:00 | 86.04 | 100.00 | 89.61 | 100.00 | 95.03 | 100.00 | 91.74 | 100.00 |

**Table 3S:** Diurnal variation of cumulative transpiration rate in *Saccharum officinarum* ‘GT 42’ plants grown under different soil moisture capacity and silicon.

| Time  (h) | Transpiration rate (mmol CO_2_ m^-2^s^-1^) irrigation level (100-95% of FC) with Si (mg L^-1^) | | | | | | | |
| --- | --- | --- | --- | --- | --- | --- | --- | --- |
|  | Cumulative | Cumulative (%) | Cumulative | Cumulative (%) | Cumulative | Cumulative (%) | Cumulative | Cumulative (%) |
|  | 0 |  | 100 |  | 300 |  | 500 |  |
| 5:00 | 0.00 | - | 0.00 | - | 0.00 | - | 0.00 | - |
| 7:00 | 1.91 | 7.64 | 2.11 | 7.81 | 2.59 | 8.61 | 2.30 | 8.08 |
| 9:00 | 6.55 | 26.10 | 7.07 | 26.18 | 8.16 | 27.14 | 7.48 | 26.27 |
| 11:00 | 12.32 | 49.30 | 13.21 | 48.91 | 14.84 | 49.35 | 13.83 | 48.58 |
| 13:00 | 17.62 | 70.51 | 18.90 | 69.97 | 21.04 | 69.97 | 19.78 | 69.48 |
| 15:00 | 21.85 | 87.43 | 23.51 | 87.04 | 26.13 | 86.90 | 24.68 | 86.69 |
| 17:00 | 24.99 | 100.00 | 27.01 | 100.00 | 30.07 | 100.00 | 28.47 | 100.00 |
| Irrigation level (80-75% of FC) with Si (mg L^-1^) | | | | | | | | |
| 5:00 | 0.00 | - | 0.00 | - | 0.00 | - | 0.00 | - |
| 7:00 | 2.08 | 9.22 | 2.12 | 9.09 | 2.16 | 8.53 | 2.09 | 8.53 |
| 9:00 | 6.36 | 28.19 | 6.48 | 27.79 | 6.87 | 27.14 | 6.66 | 27.18 |
| 11:00 | 11.37 | 50.40 | 11.63 | 49.87 | 12.54 | 49.55 | 12.15 | 49.59 |
| 13:00 | 15.94 | 70.66 | 16.37 | 70.20 | 17.77 | 70.21 | 17.21 | 70.24 |
| 15:00 | 19.68 | 87.23 | 20.28 | 86.96 | 22.03 | 87.04 | 21.34 | 87.10 |
| 17:00 | 22.56 | 100.00 | 23.32 | 100.00 | 25.31 | 100.00 | 24.50 | 100.00 |
| Irrigation level (55-50% of FC) with Si (mg L^-1^) | | | | | | | | |
| 5:00 | 0.00 | - | 0.00 | - | 0.00 | - | 0.00 | - |
| 7:00 | 1.87 | 9.63 | 1.94 | 9.53 | 2.07 | 9.65 | 2.06 | 9.86 |
| 9:00 | 5.54 | 28.54 | 5.77 | 28.34 | 5.97 | 27.82 | 5.90 | 28.24 |
| 11:00 | 9.79 | 50.44 | 10.22 | 50.20 | 10.51 | 48.97 | 10.34 | 49.50 |
| 13:00 | 13.69 | 70.53 | 14.31 | 70.28 | 14.79 | 68.92 | 14.50 | 69.41 |
| 15:00 | 16.90 | 87.07 | 17.70 | 86.94 | 18.47 | 86.07 | 18.03 | 86.31 |
| 17:00 | 19.41 | 100.00 | 20.36 | 100.00 | 21.46 | 100.00 | 20.89 | 100.00 |
| Irrigation level (35-30% of FC) with Si (mg L^-1^) | | | | | | | | |
| 5:00 | 0.00 | - | 0.00 | - | 0.00 | - | 0.00 | - |
| 7:00 | 1.27 | 8.05 | 1.33 | 8.06 | 1.44 | 8.23 | 1.41 | 8.23 |
| 9:00 | 4.08 | 25.86 | 4.25 | 25.74 | 4.49 | 25.67 | 4.42 | 25.79 |
| 11:00 | 7.56 | 47.91 | 7.84 | 47.49 | 8.24 | 47.11 | 8.12 | 47.37 |
| 13:00 | 10.85 | 68.76 | 11.28 | 68.32 | 11.86 | 67.81 | 11.67 | 68.09 |
| 15:00 | 13.61 | 86.25 | 14.19 | 85.95 | 14.97 | 85.59 | 14.70 | 85.76 |
| 17:00 | 15.78 | 100.00 | 16.51 | 100.00 | 17.49 | 100.00 | 17.14 | 100.00 |

**Table 4S:** Diurnal variation of cumulative stomatal conductance in *Saccharum officinarum* ‘GT 42’ plants grown under different soil moisture capacity and silicon.

| Time  (h) | Stomatal conductance (mmol H_2_O m^-2^s^-1^) irrigation level (100-95% of FC) with Si (mg L^-1^) | | | | | | | |
| --- | --- | --- | --- | --- | --- | --- | --- | --- |
|  | Cumulative | Cumulative (%) | Cumulative | Cumulative (%) | Cumulative | Cumulative (%) | Cumulative | Cumulative (%) |
|  | 0 |  | 100 |  | 300 |  | 500 |  |
| 5:00 | 0.00 | - | 0.00 | - | 0.00 | - | 0.00 | - |
| 7:00 | 45.39 | 3.35 | 50.62 | 3.50 | 72.78 | 4.13 | 64.78 | 4.23 |
| 9:00 | 288.43 | 21.30 | 320.90 | 22.18 | 403.59 | 22.91 | 356.28 | 23.27 |
| 11:00 | 672.70 | 49.69 | 739.78 | 51.13 | 892.56 | 50.67 | 783.07 | 51.15 |
| 13:00 | 1008.94 | 74.52 | 1096.20 | 75.77 | 1315.03 | 74.66 | 1148.63 | 75.03 |
| 15:00 | 1229.03 | 90.78 | 1322.41 | 91.4 | 1596.38 | 90.63 | 1390.16 | 90.81 |
| 17:00 | 1353.90 | 100.00 | 1446.78 | 100.00 | 1761.45 | 100.00 | 1530.89 | 100.00 |
| Irrigation level (80-75% of FC) with Si (mg L^-1^) | | | | | | | | |
| 5:00 | 0.00 | - | 0.00 | - | 0.00 | - | 0.00 | - |
| 7:00 | 43.82 | 4.09 | 57.60 | 4.90 | 75.63 | 5.65 | 60.33 | 4.75 |
| 9:00 | 248.32 | 23.20 | 295.32 | 25.12 | 352.26 | 26.29 | 314.64 | 24.77 |
| 11:00 | 550.29 | 51.41 | 624.98 | 53.16 | 719.10 | 53.67 | 671.21 | 52.84 |
| 13:00 | 807.29 | 75.41 | 897.87 | 76.38 | 1021.92 | 76.28 | 967.87 | 76.19 |
| 15:00 | 974.67 | 91.04 | 1074.31 | 91.38 | 1221.56 | 91.18 | 1160.00 | 91.32 |
| 17:00 | 1070.48 | 100.00 | 1175.59 | 100.00 | 1339.76 | 100.00 | 1270.27 | 100.00 |
| Irrigation level (55-50% of FC) with Si (mg L^-1^) | | | | | | | | |
| 5:00 | 0.00 | - | 0.00 | - | 0.00 | - | 0.00 | - |
| 7:00 | 32.98 | 4.42 | 36.93 | 4.59 | 44.42 | 4.65 | 40.94 | 4.72 |
| 9:00 | 178.74 | 23.93 | 194.42 | 24.14 | 227.31 | 23.78 | 210.06 | 24.21 |
| 11:00 | 388.49 | 52.02 | 418.60 | 51.97 | 487.88 | 51.03 | 449.04 | 51.75 |
| 13:00 | 565.34 | 75.70 | 608.11 | 75.51 | 712.51 | 74.53 | 652.23 | 75.17 |
| 15:00 | 680.55 | 91.13 | 732.78 | 90.98 | 864.53 | 90.43 | 787.57 | 90.77 |
| 17:00 | 746.78 | 100.00 | 805.39 | 100.00 | 955.99 | 100.00 | 867.65 | 100.00 |
| Irrigation level (35-30% of FC) with Si (mg L^-1^) | | | | | | | | |
| 5:00 | 0.00 | - | 0.00 | - | 0.00 | - | 0.00 | - |
| 7:00 | 29.46 | 5.48 | 35.76 | 5.85 | 43.94 | 6.44 | 36.65 | 5.55 |
| 9:00 | 124.66 | 23.19 | 154.32 | 25.23 | 175.72 | 25.75 | 162.24 | 24.56 |
| 11:00 | 255.99 | 47.62 | 312.16 | 51.03 | 346.53 | 50.77 | 332.65 | 50.35 |
| 13:00 | 378.08 | 70.33 | 449.86 | 73.55 | 497.21 | 72.85 | 482.85 | 73.08 |
| 15:00 | 471.89 | 87.78 | 548.07 | 89.60 | 607.94 | 89.07 | 590.69 | 89.41 |
| 17:00 | 537.60 | 100.00 | 611.66 | 100.00 | 682.53 | 100.00 | 660.69 | 100.00 |
